# One-Year Trajectory of Step Counts and Weight Loss in Adults With Overweight/Obesity: Retrospective Cohort Study

**DOI:** 10.2196/80339

**Published:** 2026-05-04

**Authors:** Kenshiro Taguchi, Asuka Oyama, Jun'ichi Kotoku, Hiroshi Toki, Ryohei Yamamoto

**Affiliations:** 1 Graduate Degree Program of Health Data Science, Teikyo University, 2-11-1 Kaga, Itabashi, 173-8605, Japan, 81 339641211 ext 41601; 2 Health and Counseling Center, The University of Osaka, Toyonaka, Japan; 3 Fundamental Technology Research Department, Biochemical Research Laboratory, Eiken Chemical Co, Ltd, Nogi, Japan; 4 Epidemiology Section, Division of Public Health, Osaka Institute of Public Health, Osaka, Japan; 5 Graduate School of Medical Care and Technology, Teikyo University, Itabashi, Japan; 6 Department of Nephrology, Graduate School of Medicine, The University of Osaka, Suita, Japan; 7 Laboratory of Behavioral Health Promotion, Department of Health Promotion, Graduate School of Medicine, The University of Osaka, Suita, Japan; 8 Institute for Sports and Global Health, The University of Osaka, Suita, Japan

**Keywords:** latent class mixed models, mHealth app, step counts, trajectory analysis, weight loss, physical activity, mobile health, smartphone, mobile phone, exercise, app, mHealth, observational study, Osaka, Japan, healthy lifestyle, lifestyle-related disease

## Abstract

**Background:**

Being overweight and obese are major health concerns worldwide, contributing to lifestyle-related diseases such as hypertension, dyslipidemia, type 2 diabetes, and cardiovascular disease. Increasing physical activity is an effective strategy for weight management. However, earlier step count studies have remained limited to small populations, short-term measurements of 1‐2 weeks, and mainly cross-sectional comparisons of average step counts. The effects of long-term step count changes on weight loss remain unclear.

**Objective:**

This study was conducted to assess the effects of long-term patterns of step counts on weight loss using data from the “Asmile” mobile health app in Japan. We hypothesized that participants with continuously increasing step counts over time would have a higher likelihood of significant weight reduction than participants who show steady or fluctuating patterns, even if their average step counts were similar.

**Methods:**

We analyzed data of 2778 Asmile users aged 40‐74 years with BMI ≥25 kg/m² who underwent a specific health checkup during fiscal years 2019‐2023 and who had valid step count records for 10‐14 months. Step count trajectories, reflecting long-term trends in physical activity, were classified using a latent class mixed model into four patterns: *UP* (increasing), *FLAT* (steady), *DOWN* (decreasing), and *UP/DOWN* (increasing then decreasing). Logistic regression was applied to estimate odds ratios for achieving ≥3% weight loss, with step trajectory as the explanatory variable and weight loss as the outcome.

**Results:**

Among participants, 1601 (57.6%) were men and 1177 (42.4%) were women, with respective mean ages of 65.8 (SD 7.9) and 64 (SD 8.2) years. Step count trajectories were distributed as 28.5% *UP*, 36.2% *FLAT*, 20.1% *DOWN*, and 15.2% *UP/DOWN*. Compared with the *FLAT* group, participants in the *UP* group had a significantly higher likelihood of achieving ≥3% weight loss (adjusted odds ratio 2.45, 95% CI 1.78‐3.38).

**Conclusions:**

Long-term tracking of step counts using the Asmile app revealed distinct activity patterns. Continuous increases in step counts were associated with the greatest likelihood of weight loss, emphasizing the importance of sustained physical activity. These findings support the use of long-term step monitoring to guide interventions for obesity and lifestyle-related disease prevention.

## Introduction

Being overweight and obese have come to pose medical and social problems in economically advanced countries, including Japan, and in some low- and middle-income countries [1-4]. According to the World Health Organization, which provides widely accepted global guidelines, people with a BMI of 25 kg/m² or greater are classified as overweight, whereas those with a BMI of 30 kg/m² or greater are classified as obese [4]. Obesity is associated with lifestyle-related diseases such as hypertension [5], dyslipidemia [6], type 2 diabetes [7], and arteriosclerotic cardiovascular disease (CVD) [8]. In Japan, the Japan Society for the Study of Obesity in 2000 defined obesity as having a BMI greater than 25 kg/m^2^ [9]. To avoid confusion caused by different definitions among countries, we designate both being overweight and being obese as “obesity” throughout this paper. For 3480 Japanese people with obesity or metabolic syndrome, Muramoto et al [10] reported that achievement of 3% weight loss improves risk factors associated with obesity. In obese populations, promoting weight loss is useful and important to reduce the risks of developing lifestyle-related diseases. Therefore, weight loss is often necessary to maintain health.

Walking is a daily physical activity that promotes weight loss and improves lifestyle-related diseases. Pedometers have been used for objective measurement of an individual’s per-day step count. Earlier cross-sectional studies comparing BMI and average daily step counts have shown that individuals with higher step counts tend to have lower body weight than those with fewer steps [11-13]. A negative correlation was found between short-term physical activity and physical status [14]. However, earlier studies using pedometers have been burdened by several limitations: small sample sizes and short-term monitoring periods, typically 1‐2 weeks. Moreover, although a negative correlation has been found, those results derive only from cross-sectional analyses. They do not reflect changes in step counts and weight loss induced by these changes. Therefore, relations between long-term and longitudinal step count changes and weight loss remain unclear.

For this study, we applied trajectory analysis to assess long-term changes in step counts. Latent class mixed models (LCMMs), a trajectory analysis approach, can cluster heterogeneous populations into latent and more homogeneous trajectories [15]. They are then classifiable by group, reflecting different long-term changes. Such models have been applied to the identification of long-term clinical phenotypic changes and elucidation of their group characteristics because skin-thickening trajectories are associated with organ involvement and survival [16]. The trajectories of systemic lupus erythematosus support the feasibility of performing adaptive trial designs [17]. Therefore, by analyzing the trajectories as variables reflecting homogeneous changes over a long period, one can better understand the near-causal effects on outcomes from those trajectories [18].

Therefore, this study was conducted to elucidate the effects of long-term fluctuations in step counts on weight reduction. From this observational study, we obtained approximately 1 year of step count data from 2778 users of the “Asmile” mobile health care (mHealth) app. Users were classified based on their step count trajectories inferred using LCMM. We hypothesized that, rather than simply comparing average step counts, long-term walking patterns would influence subsequent weight loss: specifically, participants with a continuous increase in step counts, rather than a temporary increase, would be more likely to achieve significant weight reduction.

## Methods

### Mobile Health Care App “Asmile”

The Asmile mHealth app, available on iOS and Android, was released in 2019. It is provided by the Osaka Prefectural Government [19]. As of May 2025, more than 450,000 Asmile users have adopted this app to promote their health by recording daily activities and facilitating self-monitoring. One Asmile feature allows a user to record the walking step count automatically as a daily activity by integration with standard smartphone health care apps. Furthermore, daily user information (weight, body temperature, etc) and lifestyle habits (sleeping hours, breakfast intake, tooth brushing, etc) can be recorded manually in the Asmile app. Users with healthy lifestyles, such as those who walk a lot, are awarded points, which they can use to enter drawings for prizes such as electronic money. The granting of such digital incentives encourages more walking [20,21].

Another feature is that results of specific health checkups (SHCs) for National Health Insurance subscribers can be synchronized automatically with the Asmile app. This SHC is aimed at primary prevention and early detection of obesity and lifestyle-related diseases for all insured persons aged 40‐74 years [22-24]. SHC data such as height, weight, BMI, blood test data, and urinalysis data are recorded. Therefore, the Asmile app allows users to check daily activity records over the long term and to check their health status by fiscal year. Using the Asmile app promotes healthy daily activities and increases awareness of one’s health, which is expected to prevent lifestyle-related diseases.

Furthermore, recent usage of the Asmile app has revealed effects of the declaration of a state of emergency for COVID-19 on smoking behavior and the relation between oral frailty and falls [25,26], in addition to a causal effect on increased step counts [27]. Utilization promotes healthy daily activities and increases awareness of one’s health, both of which are expected to prevent lifestyle-related diseases.

### Participants

This study included participants who registered for the Asmile app during fiscal years 2019‐2023 and who underwent their first SHC after registration and before the end of fiscal year 2023. We excluded participants with a BMI of less than 25 kg/m². In addition, because this study specifically addressed changes in step count and weight over a period of approximately 1 year, we excluded users who did not undergo a second SHC 10‐14 months after the first SHC. For participants with more than one eligible SHC pair, we selected the SHC pair for which the interval between the first and second SHC was closest to 12 months.

**Figure 1.**
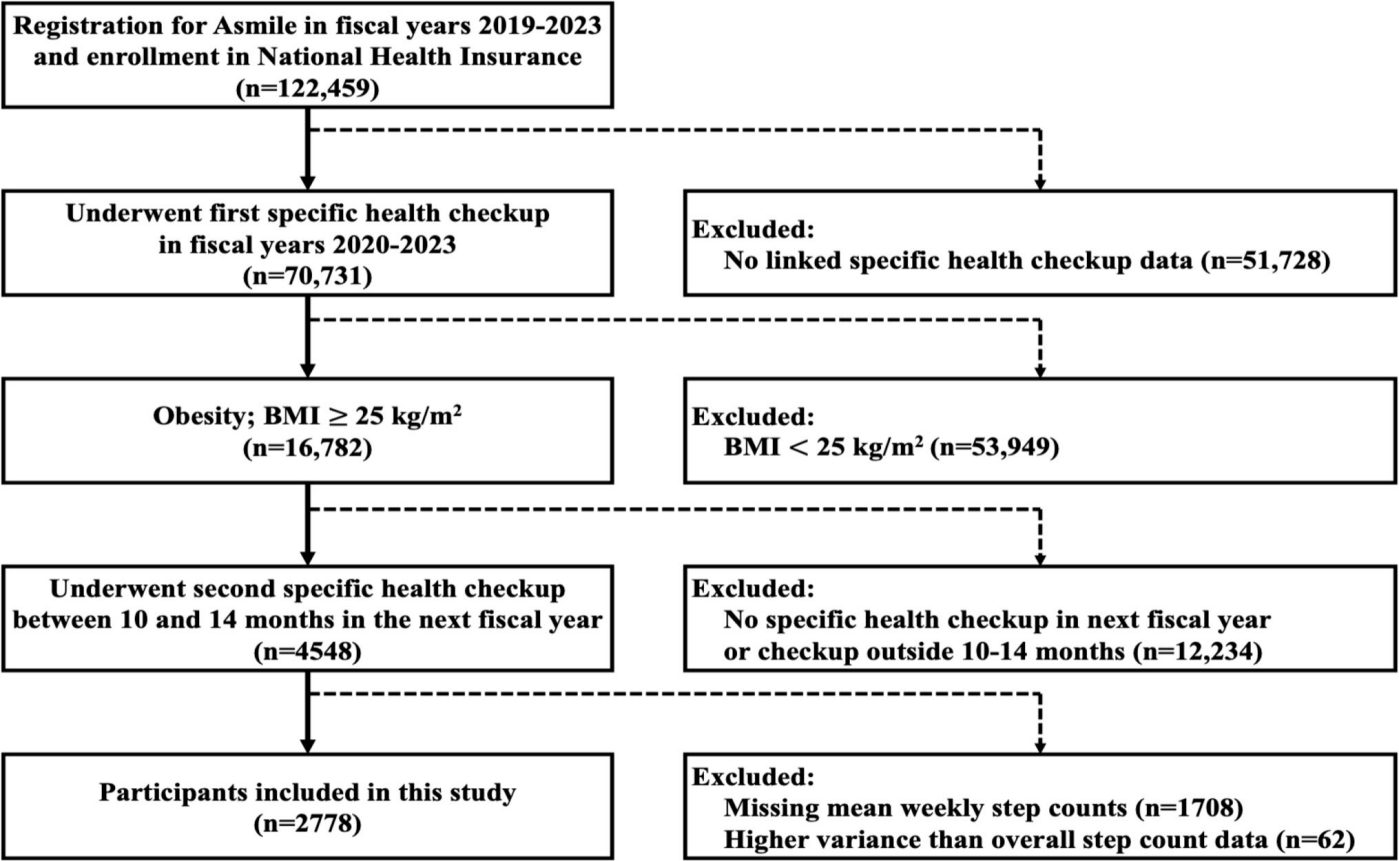
Flowchart of the included participants’ selection for analysis in this study.

In total, data from 122,459 users enrolled in the National Health Insurance and newly registered for the Asmile app were screened. Among those users, 70,731 underwent the first SHC during the 2019‐2023 fiscal years. After application of the exclusion criteria, 2778 participants with complete step count records and SHC links were included in the analysis (Figure 1).

### Step Count Data

Step count data were recorded in the Asmile app by automatic integration with standard smartphone health care apps. To provide additional detail, the daily step count for the last 42 days was transferred each time the user opened the Asmile app. Considering misplaced or forgotten smartphone or system inadequacies, inappropriate step count data of fewer than 200 steps and greater than 50,000 steps per day were excluded. Those cutoff values were inferred based on the step count distribution of Asmile users [27]. We used step count data with larger steps in a day, for which the data were recorded using both iOS and Android.

When preprocessing the step count data, we first excluded participants without step count data for at least 1 day per week, and those with a higher variance than all step count data. Then, we calculated the weekly average step count for each week from the first SHC until the second SHC. In all, 2778 participants were included in the analysis, with no missing values.

### Latent Class Mixed Models

For this study, we used an LCMM [15] to identify long-term step trajectories of Asmile users. This approach, assuming that there are *G* latent classes in a heterogeneous population, can classify them into *G* trajectories. This method accommodates the identification of long-term changes in physical activity from longitudinal step count data.

Letting *N* represent the number of individuals $i\in \left\{1,\ldots ,N\right\}$, with step count observations *Y_ij_* at occasion *j*, we aim to classify these individuals into latent classes $g\in \left\{1,\ldots ,G\right\}$, each representing characteristic patterns such as increasing, decreasing, flat, or non-monotonic trajectories over time. The step count model is


(1) $$\begin{array}{c} Y_{ij|g}=\beta_{0g}+\beta_{1g}t_{ij}+\beta_{2g}t_{ij}^{2}+u_{ig}+\epsilon_{ij}\; \end{array}$$

In the above equation, *t_ij_* stands for the elapsed weeks at occasion *j*, *β* represents the fixed effects of latent class *g*, and *u_ig_* denotes the random effects for participant *i* of latent class *g* that is independent of time. In addition, measurement error $\epsilon_{ij}$ follows a Gaussian distribution of mean 0 and variance $\sigma_{\epsilon }^{2}$. The LCMM approach estimates model parameters by maximizing the marginal log-likelihood [28] as


(2) $$\begin{array}{c} l\left(\theta \right)=\sum_{i=1}^{N}\log\left(\sum_{g=1}^{G}\pi_{g}L_{g}\left(Y_{i}|t_{i},\;\theta \right)\right) \end{array}$$

In the equation, *θ* denotes the set of parameters to be estimated; $\pi_{g}$ represents the prior probability of latent class *g* as


(3) $$\begin{array}{c} \pi_{g}=\frac{e^{\gamma_{g}}}{\sum_{l=1}^{G}e^{\gamma_{l}}} \end{array}$$

where $\gamma_{g}$ is a class-specific intercept-like parameter. Because the step counts are typically approximately 5000, we assume a multivariate normal distribution for the likelihood function $L_{g}\left(Y_{i}|t_{i},\theta \right)$. The posterior probability that individual *i* belongs to class *g* is given as


(4) $${\hat{\pi }}_{ig}=\frac{\pi_{g}\;L_{g}\;\left(y_{i}|t_{i},\theta \right)}{\sum_{l=1}^{G}\pi_{l}\;L_{l}\;\;\left(y_{i}|t_{i},\theta \right)}$$

In practice, the log-likelihood is then maximized iteratively using a modified Marquardt iterative algorithm and the Newton-Raphson method. Each individual is assigned to the latent class *g* for which the posterior probability ${\hat{\pi }}_{ig}$ is the highest.

Next, as one condition for determining the number of latent classes, we obtained ${\overset{-}{\pi }}_{g}$: the mean of the posterior probabilities of belonging to the latent class among the participants. We determined *G* (the number of latent classes) under the following three conditions. First, a participant *i* belongs to latent class *g* that has the largest ${\hat{\pi }}_{ig}$. At this point, we ensured that the number of participants belonging to each latent class constituted at least 5% of the total number of participants analyzed. Second, the average posterior probability ${\overset{-}{\pi }}_{g}$ in each latent class was set as approximately 90% or higher. Third, we selected the optimal *G* (number of latent classes) with the minimum Bayesian information criterion under conditions 1 and 2.

We used the lcmm package [29] with R software to identify step trajectories. Earlier reports [15,30] provide additional details related to the LCMM algorithm.

### Statistical Analysis

Categorical variables were expressed as numbers and proportions, whereas continuous variables were expressed as the mean (SD). The means of step counts were calculated for three periods: from undergoing the first SHC to undergoing the second SHC, from undergoing the first SHC to 28 elapsed weeks, and from 28 elapsed weeks to undergoing the second SHC (maximum 56 elapsed weeks). Differences in these periods’ associated variables were analyzed using *t* tests for men and women, analysis of variance between latent classes, and the chi-square test for categorical variables.

Long-term step counts of Asmile users were classified as step trajectories using LCMM. All Asmile users belonged to one of the *g* latent classes. We performed logistic regression using the *g* latent classes classified by LCMM as explanatory variables. The response variable was the presence or absence of weight loss of 3% or more at the second SHC, around one year after the start of step count recording, because achievement of 3% weight loss improves risk factors associated with obesity [10]. Adjustment variables included age, sex, mean step counts, smoking status, and the presence or absence of drug history. Here, drug history refers to whether a participant reported, at the time of the health checkup, taking medication for one or more of the following: diabetes, hypertension, or dyslipidemia. Specific drug names or classes, such as GLP-1 RAs or SGLT2 inhibitors, were not available. The odds ratios for each step trajectory were estimated. For sensitivity analysis, odds ratios were estimated for 1006 Asmile users who were not taking medication, to exclude the possibility of hospital attendance. The *P* values of the estimated partial regression coefficients are based on the Wald statistic.

Here, *P*<.05 was inferred as significant. All statistical analyses were conducted using R (version 4.3.1) with the lcmm package (version 2.1.0). This study is reported in accordance with the STROBE (Strengthening the Reporting of Observational Studies in Epidemiology) guidelines.

### Ethical Considerations

The study protocol was approved by the ethics committee of the Health and Counseling Center of The University of Osaka (institutional review board approval number 8 in 2024). All procedures involving human participants were conducted according to the 1964 Declaration of Helsinki and its later amendments or comparable ethical standards. At the time of app registration, the Asmile users consented to the use of nonidentifiable information in accordance with the terms of service related to the privacy policy. Informed consent to this study from the participants was waived because all data were anonymized according to the Japanese Ethical Guidelines for Medical and Health Research Involving Human Subjects enacted by Japan’s Ministry of Health, Labour and Welfare. Anonymized data were provided by the Osaka Prefectural Government. No compensation was provided to participants.

## Results

### Characteristics

Table 1 presents baseline characteristics of the 2778 users. For weight and BMI, the first SHC was defined as preweight and pre-BMI. The second SHC was defined as postweight and post-BMI. Of the participants, 1601 (57.6%) were men and 1177 (42.4%) were women, with respective mean ages of 65.8 (SD 7.9) and 64 (SD 8.2) years. The mean step counts were significantly different between men and women, with men averaging 6461 (SD 3481) steps/day and women averaging 4953 (SD 2483) steps/day (*P*<.001). In addition, men and women had different weights (*P*<.001). Nevertheless, no difference was found for either BMI (pre-BMI: *P*=.18; post-BMI: *P*=.84) or weight difference after about 1 year (*P*=.41).

**Table 1.** Baseline characteristics.

Characteristics	Overall (n=2778)	Men (n=1601)	Women (n=1177)	*P* value
Age (years), mean (SD)	65.0 (8.1)	65.8 (7.9)	64.0 (8.2)	<.001
Elapsed weeks, mean (SD)	52.1 (3.4)	52.1 (3.4)	52.1 (3.4)	.76
Step counts, mean (SD)	5822.1 (3185.6)	6460.9 (3481.2)	4953.2 (2482.9)	<.001
Preweight^a^, mean (SD)	72.1 (9.8)	76.9 (8.7)	65.6 (7.2)	<.001
Postweight^a^, mean (SD)	71.3 (10.1)	76.1 (8.9)	64.7 (7.6)	<.001
Weight difference, mean (SD)	−0.8 (3.0)	−0.8 (2.9)	−0.9 (3.1)	.41
Weight loss rate, mean (SD)	−1.2 (4.0)	−1.0 (3.6)	−1.3 (4.5)	.04
Pre-BMI^a^, mean (SD)	27.2 (2.3)	27.2 (2.3)	27.3 (2.4)	.18
Post-BMI^a^, mean (SD)	26.9 (2.5)	26.9 (2.4)	26.9 (2.6)	.84
Smoking status, n (%)				<.001
No	2569 (92.5)	1427 (89.1)	1142 (97.0)	
Yes	209 (7.5)	174 (10.9)	35 (3.0)	
Medication status, n (%)				<.001
No	1006 (36.2)	529 (33.0)	477 (40.5)	
Yes	1772 (63.8)	1072 (67.0)	700 (59.5)	

a Preweight and pre-BMI were defined as measurements obtained at the first specific health checkup (SHC). Postweight and post-BMI as those obtained at the second SHC.

### Classification of Step Count Trajectory

**Figure 2.**
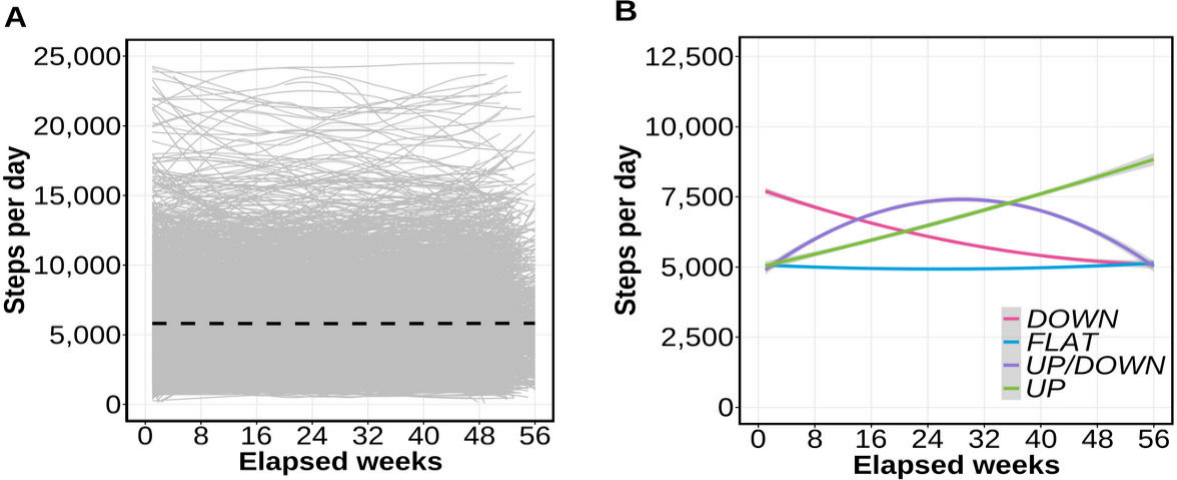
Results of step trajectories. Horizontal axis: number of weeks elapsed from undergoing the first SHC to the second SHC. Vertical axis: average steps per day at each elapsed week. (A) All individual trajectories and the average trend using B-splines. Gray solid line: step trajectories for each of the 2778 individuals. Black dotted line: average step trajectory of 2778 participants. (B) Results of the latent 4 classes as step trajectories classified by the step count model using latent class mixed models. SHC: specific health checkup.

Figure 2 shows each trajectory of the 2778 participants and notable heterogeneity among participants. Models with 1‐5 latent classes were built sequentially. Under conditions in which at least 5% of 2778 participants belonged to each latent class with at least a 90% probability, the 4-class model had the lowest Bayesian information criterion (Multimedia Appendices 1-3). That finding confirmed that assuming 4 classes provided the best explanatory power. Actually, the 4 classified step trajectories reflected walking styles over the long term. We labeled these trajectories as *UP*, *DOWN*, *UP/DOWN*, and *FLAT*.

Table 2 presents characteristics of the 4 latent classes (*UP*, *DOWN*, *UP/DOWN*, and *FLAT*). Among the classes, age (*P*=.82) and the duration until undergoing the next checkup (*P*=.07) were found to have no significant difference. In addition, no significant difference was found for any class in pre-BMI (*P*=.39), but a reduction in BMI for the *UP* class was found in comparison to the other classes for post-BMI (*P*=.01). The average step count of the *FLAT* class was low compared to the other classes (*P*<.001). More notably, irrespective of the long-term step count data, the average physical activities of the *UP*, *DOWN*, and *UP/DOWN* classes were almost identical when compared simply: 7592 (SD 3302) steps/day, 7223 (SD 3852) steps/day, and 7541 (SD 3282) steps/day.

**Table 2.** Characteristics of the 4 classes as step trajectories.

Characteristic	Overall (n=2778)	DOWN (n=355)	UP/DOWN (n=193)	FLAT (n=2045)	UP (n=185)	*P* value
Gender, n (%)						<.001
Men	1601 (57.6)	223 (62.8)	134 (69.4)	1125 (55.0)	119 (64.3)	
Women	1177 (42.4)	132 (37.2)	59 (30.6)	920 (45.0)	66 (35.7)	
Age (year), mean (SD)	65.0 (8.1)	64.6 (8.2)	65.1 (8.7)	65.1 (8.0)	65.1 (8.3)	.82
Elapsed weeks, mean (SD)	52.1 (3.4)	51.2 (3.4)	52.62 (3.3)	52.0 (3.4)	52.3 (3.5)	.07
Step counts, mean (SD)	5822.1 (3185.6)	7223.4 (3851.8)	7541.0 (3282.3)	5256.5 (2828.6)	7591.5 (3301.8)	<.001
Steps up to 28 weeks, mean (SD)	5826.3 (3227.1)	8023.8 (3951.5)	7347.1 (3304.2)	5243.2 (2828.2)	6469.0 (3309.3)	<.001
Steps after 29 weeks, mean (SD)	5813.5 (3248.1)	6278.0 (3801.5)	7763.0 (3345.3)	5270.2 (2863.5)	8894.1 (3381.3)	<.001
Preweight^a^, mean (SD)	72.1 (9.8)	72.5 (10.0)	74.5 (10.0)	71.9 (9.8)	72.2 (9.0)	.004
Postweight^a^, mean (SD)	71.3 (10.1)	72.0 (10.6)	73.6 (10.5)	71.1 (10.1)	70.1 (9.1)	.002
Weight difference, mean (SD)	−0.8 (3.0)	−0.5 (2.7)	−0.9 (3.5)	−0.8 (2.9)	−2.1 (3.4)	<.001
Weight loss rate, mean (SD)	−1.2 (4.0)	−0.7 (3.8)	−1.3 (4.6)	−1.1 (3.9)	−2.8 (4.7)	<.001
Pre-BMI^a^, mean (SD)	27.2 (2.3)	27.1 (2.1)	27.4 (2.3)	27.2 (2.4)	27.1 (2.0)	.39
Post-BMI^a^, mean (SD)	26.9 (2.5)	26.9 (2.4)	27.1 (2.6)	27.0 (2.5)	26.3 (2.3)	.010
Smoking status, n (%)						.29
No	2569 (92.5)	337 (94.9)	177 (91.7)	1883 (92.1)	172 (93.0)	
Yes	209 (7.5)	18 (5.1)	16 (8.3)	162 (7.9)	13 (7.0)	
Medication status, n (%)						.11
No	1006 (36.2)	145 (40.8)	78 (40.4)	719 (35.2)	64 (34.6)	
Yes	1772 (63.8)	210 (59.2)	115 (59.6)	1326 (64.8)	121 (65.4)	
Outcome, n (%)						<.001
Weight loss <3%	2123 (76.4)	280 (78.9)	146 (75.6)	1588 (77.7)	109 (58.9)	
Weight loss ≥3%	655 (23.6)	75 (21.1)	47 (24.4)	457 (22.3)	76 (41.1)	

a Preweight and pre-BMI were defined as the measurements obtained at the first specific health checkup (SHC). Postweight and post-BMI were defined as those obtained at the second SHC.

**Figure 3.**
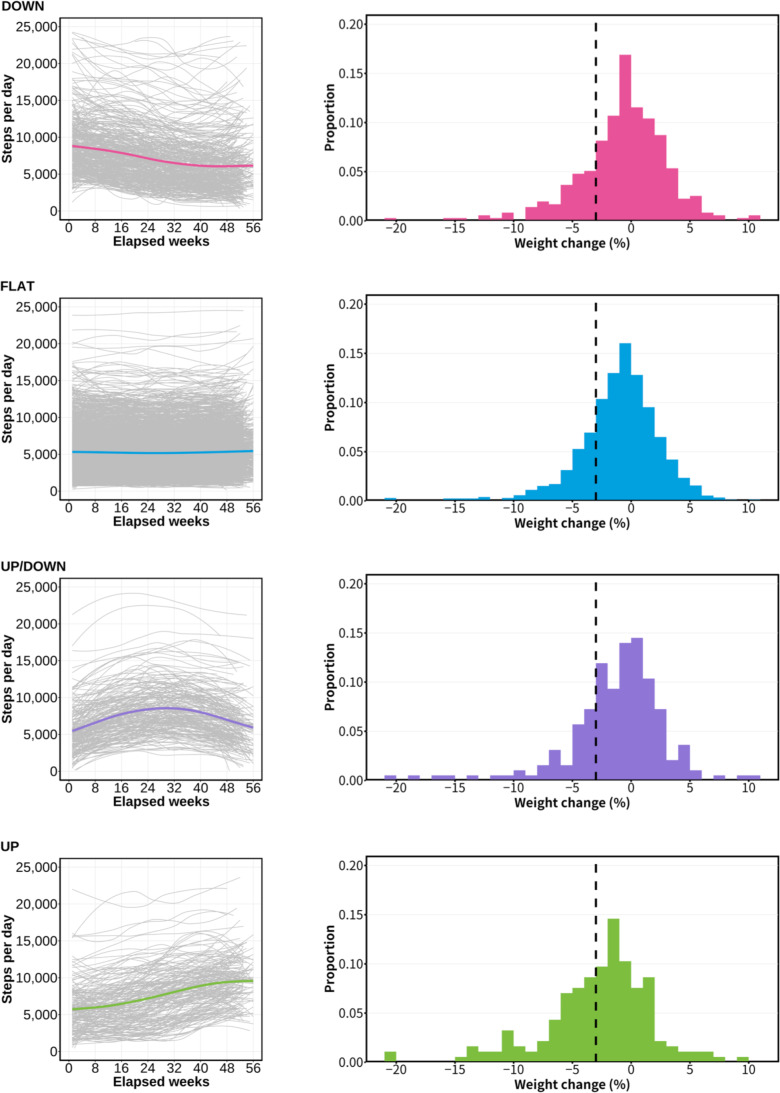
Comparison of step trajectory and weight changes among the 4 latent classes. (Left panels) Individual step trajectories and the average trend using B-splines in each latent class. Gray solid line: walking trends for individuals in each latent class. Each color line: average step trajectory in each latent class. (Right panels) Distribution by weight changes about 1 year in each latent class. Horizontal axis: weight change in increments of 1%, rounded below −20% and above 10%. Vertical axis: percentage of people in the latent class who have achieved a certain weight change after about 1 year. Black dotted line: 3% weight loss target cutoff used for this study. Latent classes from (top panels) to (bottom panels): *DOWN*, *FLAT*, *UP/DOWN*, and *UP*.

Figure 3 shows trajectories of the 4 latent classes (*UP*, *DOWN*, *UP/DOWN*, and *FLAT*) and the weight changes in the respective classes. *UP*, *DOWN*, *UP/DOWN*, and *FLAT* exhibited long-term step changes with the respective trends of increasing, decreasing, increasing and decreasing, and steady. It is noteworthy that the *UP* class included the most cases of more than 3% weight loss. The distribution of weight change was symmetrical in *FLAT*. In *DOWN*, compared to *FLAT*, the proportion with weight loss of around 3% was lower, whereas the proportion with weight gain of 5% or more was higher. The distribution in the *UP*/*DOWN* class was polarized. Among all classes, the *UP* class showed the highest proportion of users with weight loss of 3% or more.

The *UP* class was characterized by a long-term increase in step counts: the mean of step counts for 1‐28 elapsed weeks was 6469 (SD 3309) steps/day, but after 29 elapsed weeks, it was 8894 (SD 3381) steps/day. This class included 185 participants, 76 (41.1%) of whom were found to have greater than 3% weight loss. The absolute weight change was −2.1 (SD 3.4) kg. The relative weight change was −2.8% (SD 4.7%). These were the greatest negative values among all classes.

The *DOWN* class was characterized by a long-term decrease in step counts: the mean step count for 1‐28 elapsed weeks was 8024 (SD 3952) steps/day; that after 29 elapsed weeks was 6287 (SD 3802) steps/day. This class included 355 participants, 75 (21.1%) of whom achieved greater than 3% weight loss. The preweight and postweight differences and weight loss were, respectively, −0.5 (SD 2.7) kg and −0.7% (SD 3.8%): the largest values among all classes.

The *UP/DOWN* class was characterized by a long-term increase and decrease in step counts: the mean step count for 1‐28 elapsed weeks was 7347 (SD 3304) steps/day; after 29 elapsed weeks, it was 7763 (SD 3345) steps/day. This class included 193 participants whose mean step counts of 7541 (SD 3282) steps/day were higher than the overall mean step counts of 5822 (SD 3186) steps/day and the *FLAT* group’s mean step counts of 5257 (SD 2829) steps/day.

The *FLAT* class was characterized by long-term stay-in step counts: the mean step count for 1‐28 elapsed weeks was 5243 (SD 2828) steps/day; that after 29 elapsed weeks was 5270 (SD 2864) steps/day. This class, which included 2045 participants, had mean step counts of 5257 (SD 2829) steps/day, which were closest to the overall mean step counts of 5822 (SD 3186) steps/day.

### Step Count Trajectory Effects on Weight Loss

**Figure 4.**
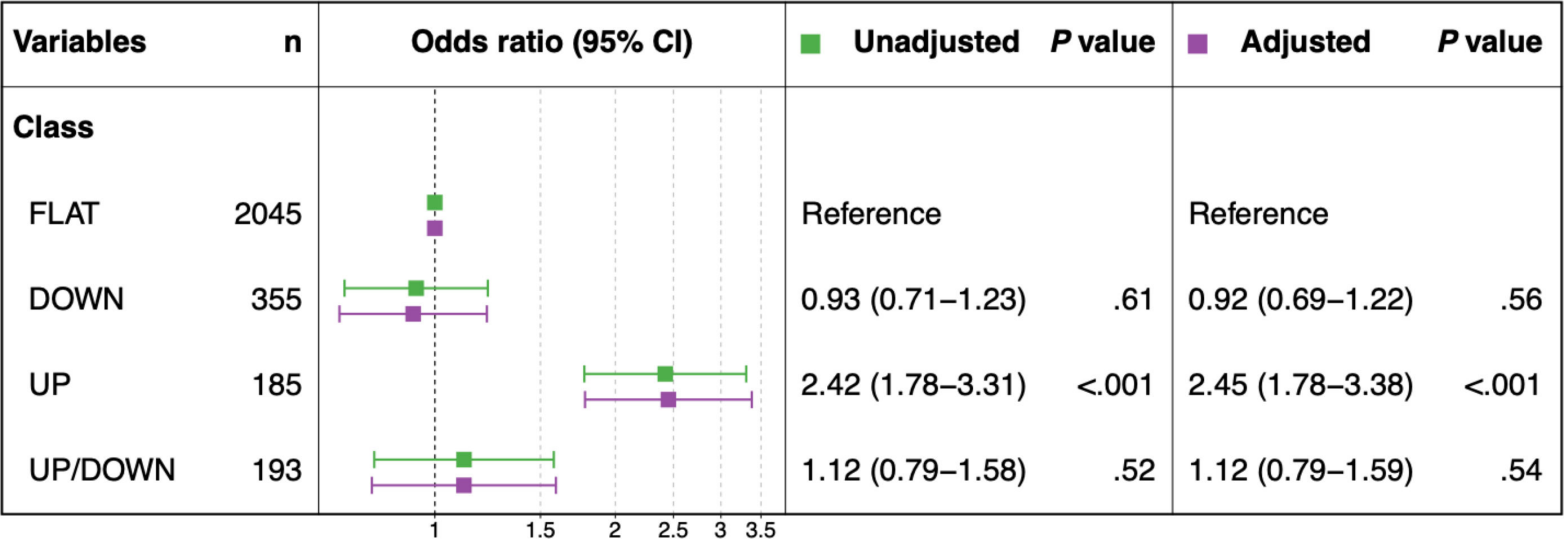
Forest plot of odds ratios of the respective trajectories for weight loss.

Figure 4 presents the estimated odds ratios of each trajectory for 3% weight loss. Performing multivariable logistic regression analysis with 4 latent classes (*UP*, *DOWN*, *UP/DOWN*, and *FLAT*), we assessed effects on weight loss from *UP*, *DOWN*, and *UP/DOWN* compared with *FLAT* (reference). The adjusted odds ratios of *UP*, *DOWN*, and *UP/DOWN* were, respectively, 2.45 (95% CI 1.78‐3.38), 0.92 (95% CI 0.69‐1.22), and 1.12 (95% CI 0.79‐1.59). Among these, a significant association was found only for the *UP* class. By contrast, no significant difference was found for any *DOWN* or *UP/DOWN* class relative to the *FLAT* class.

### Sensitivity Analysis

The effects of those step trajectories on weight loss were estimated as odds ratios for 1006 Asmile users who were taking no medication. These adjusted odds ratios of *UP*, *DOWN*, and *UP/DOWN* were, respectively, 2.42 (95% CI 1.41‐4.13), 1.07 (95% CI 0.71‐1.63), and 1.30 (95% CI 0.77‐2.19). Sensitivity analysis estimated the effects on weight loss for those who did not take medications. Results demonstrated that the effects of clusters of step counts on weight loss remained (Multimedia Appendix 4).

## Discussion

### Principal Results

This trajectory analysis of 2778 Asmile members linked to SHC confirmed the usefulness of classification into 4 latent classes of *UP*, *DOWN*, *UP/DOWN*, and *FLAT*, which respectively represented increased, decreased, increased/decreased, and unchanged step counts for 10‐14 months. Logistic regression analysis with these 4 latent classes suggested an association of a long-term increase in step counts with achieving greater than 3% weight loss, whereas a decrease in step counts was not associated significantly with weight loss about 1 year later.

Results indicated that subgroups, as latent classes, exist within the long-term step trajectory (Figure 2). We also characterized those subgroups (Table 2). These subgroups were classified as 4 heterogeneities of *UP*, *DOWN*, *UP/DOWN*, and *FLAT,* respectively, defined as described above (Figure 3).

For *UP*, the highest weight loss and the lowest post-BMI were observed. In earlier studies, a correlation between physical activity and BMI was proposed. Particularly, Krumm et al [11] and Thompson et al [12] reported that women with levels of activity such as approximately 10,000 steps/day typically have a BMI of less than 25 kg/m^2^. Dwyer et al [31] reported that, for people with low daily energy expenditure, such as <10,000 steps/day, increasing daily step counts contributes to the reduction of obesity. These results are cross-sectional analyses, particularly addressing average steps, but they reveal a negative correlation between the average steps and BMI. In contrast, our results are longitudinal analyses, particularly addressing long-term step changes. In addition, the *UP* results support their association because the increased step counts show the greatest weight loss over the long term. It is noteworthy that the results indicate long-term increased physical activity as one factor affecting weight loss.

For *DOWN*, we observed the lowest weight loss, along with higher post-BMI than *UP*. Wyatt et al [13] reported that people with a BMI greater than or equal to 30 kg/m² take 2400 steps/day fewer than those with a BMI of less than 25 kg/m². They report a negative correlation between the average steps and BMI in cross-sectional analyses, particularly addressing average steps. Additionally, for some patients with advanced cancer undergoing chemotherapy, Manz et al [32] reported that a 1000-step/day decrease is associated with a 16% higher risk of hospitalization or death. Their results revealed risks attributable to a decrease in daily steps. The *DOWN* results can support their finding of negative correlation because intermittent decreased step counts over a long term show higher post-BMI than *UP*. In fact, for almost identical average step counts reported for *UP* and *DOWN*, findings indicate that decreased physical activity over the long term might achieve only insufficient weight loss. Therefore, to reduce risks associated with the decrease in step counts, a person should strive to increase and maintain step counts over a long period.

For *UP/DOWN*, we observed higher mean step counts than for *FLAT*, indicating greater weight loss than for *FLAT*. In addition, even though the mean step counts for *UP/DOWN* were closer to those of *UP* and *DOWN*, they showed less weight loss than that achieved by *UP* and greater weight loss than that achieved by *DOWN*. This finding, which was not obtained from a simple comparison of mean step counts, suggests that increased intensity of physical activity over the long term is strongly reflected in weight loss.

For *FLAT*, which included the most Asmile users, baseline characteristics including age, sex, mean step counts, and BMI were similar to those of the overall population. Because *FLAT* reflects the distribution of the overall population in this study, we used *FLAT* as a reference when estimating weight loss effects.

To estimate the odds ratios of step trajectories for greater than 3% weight loss, we performed logistic regression analysis using the 4 latent classes: *UP*, *DOWN*, *UP/DOWN*, and *FLAT*. Actually, *UP* showed a higher adjusted odds ratio of 2.45 when using *FLAT* as a reference, including significantly more cases of weight loss greater than 3%. The results estimated a significant effect of increased step counts, reflecting long-term fluctuations of weight loss, because explanatory variables classified long-term fluctuations into several step trajectories as a latent class with LCMM. It is noteworthy that the average amounts of physical activity were similar for *UP*, *DOWN*, and *UP/DOWN*, but the *UP* group, which reflected increased and maintained step counts over the long term, was more likely to achieve weight loss than either the *DOWN* or *UP/DOWN* class.

Furthermore, most earlier studies using step counts have compared mean step counts. They have not considered long-term fluctuations in step counts, particularly capturing a decrease or a pattern of increase and decrease. From this study, *DOWN* and *UP/DOWN* were found to have no significant weight loss for *FLAT* as a reference, with respective odds ratios of 0.92 and 1.12, which are close to 1. As a notable finding, results suggest that a sustained walking style was associated more strongly with achievement of significant weight loss because the odds ratio for the overall mean step count was 1.01 per 1000-step increase, suggesting a possible, though nonsignificant, association between daily step count and weight loss. However, a higher odds ratio was observed for *UP*, which reflects a long-term walking style (Multimedia Appendix 5). These findings suggest that long-term increase and maintenance of physical activity such as walking, rather than temporary increases in physical activity, more strongly affect weight loss. In turn, this will engender a reduction in the risk of developing diseases caused by obesity.

### Comparison With Earlier Work

Several earlier studies have evaluated mHealth app effects on physical activity, but most have had limited sample sizes and comparisons of simple step counts. In their meta-analysis, Flores Mateo et al [33] and Islam et al [34] reported that interventions using the mHealth app showed greater weight loss than the control. However, no significant difference in physical activity was found. Moreover, an important limitation is that these physical activities were recorded and assessed using a questionnaire and self-reporting. The data were not recorded automatically. In a randomized controlled trial of a mobile app intervention during a longer-term 32 weeks, Yoshimura et al [35] recorded and assessed physical activities using a step-count-specific app during weight loss. The intervention group was also instructed to use the app to check their daily step counts and ranks. Particularly, they revealed effective step increases on weekends. Nevertheless, no effect on weight loss was observed. They suggested the need to develop specialized tools to enhance weight loss effects. Their study represents an excellent analysis of the long-term effects of physical activity using a step count-specific app, but it falls short as an evaluation of weight loss when considering long-term step changes.

Painter et al [36] reported self-monitoring, such as weight, step counts, and food, as significant predictors of weight loss during a 6-month intervention. Their analysis showed that the weight loss ≥10%, 5%‐10%, and <5% groups had 6-month mean step counts, respectively, of 8078, 6657, and 5277 steps/day. A significant difference was also found among those means of step counts. Their study simply compared mean step counts and weight loss, confirming their mutual relation. By contrast, our observational study revealed a relation between long-term step-count changes and weight loss. Mean step counts were nearly equivalent among the *UP*, *DOWN*, and *UP/DOWN* classes, but significant weight loss was observed only for *UP*. These results clarify the relation between step count and weight loss from different perspectives. Therefore, considering that the *UP* class, the group with increased step counts, is more likely to achieve weight loss, the results reported by Painter et al [36] are supported: groups with greater weight loss had higher mean step counts. The findings reported herein further suggest that walking style is important for achieving long-term weight loss and suggest that increasing and maintaining physical activity levels is crucially important.

### Benefits of Increasing and Maintaining Step Counts Long-Term

Oyama et al [27] reported a causal effect of using Asmile on increased step counts by approximately 400 steps/day and approximately 10,000 steps during 4 weeks in a large observational study of 80,689 Asmile users in Osaka prefecture.

The benefits of increased step counts for aiding weight loss and reducing CVD risk have been reported. In a randomized controlled trial including young adults, Rogers et al [37] analyzed step count effects on postprandial metabolism and showed that CVD risk was reduced by 10,000 steps/day, which significantly reduced postprandial lipemia, an independent predictor of CVD, compared with 2000 steps/day activity. Therefore, considering the *UP* effects of weight loss found from our study, continuous health promotion in mHealth, including Asmile, supports improved health status and prevention of lifestyle-related diseases.

### Limitations

This study has several strengths, such as its large sample size linked to continuous daily activity (long-term step counts) and health status (medical checkup for each year), the classification of individuals’ heterogeneous step count trajectories into latent classes, and the odds ratio estimation as identifying effects of long-term step count trajectories on weight loss.

However, the study also has some limitations. First, we were able to track the medication use of Asmile users (ie, whether they reported taking medications for diabetes, hypertension, or dyslipidemia), but their actual hospital attendance was unknown. These are fundamentally important unobserved confounders because they might influence physical activity and weight change. To address potential confounding by medication use, we conducted a sensitivity analysis including only participants who were not taking any medication. The results were consistent with those of the main analysis.

Second, the Asmile users’ occupational characteristics are not considered. Changes in physical activities might occur because of occupational changes; for example, white-collar workers spend more time sitting than blue-collar workers during daily life [38]. Whereas step counts provided detailed information related to daily movement, participants with 1 or more weeks in which step count data were missing completely were excluded from the analysis. In the Asmile app, daily step counts for the last 42 days are transferred each time the user opens the app. Therefore, missing data generally occur because of participants’ app usage behavior rather than because of random loss. For that reason, excluding these participants might introduce selection bias. Nevertheless, we regarded this approach as appropriate for a more conservative analysis because it allowed us to examine high-quality longitudinal step count data specifically and address an important knowledge gap related to the effects of physical activity over time on weight change.

Third, Asmile users are likely to be more health conscious than people who are not using the mHealth app, including Asmile. Therefore, caution must be exercised when roughly generalizing and interpreting the results of this analysis. Moreover, because the study population consisted mainly of active individuals with a BMI ≥25 kg/m² who participated in the app-based program voluntarily, the findings might not be generalizable to individuals with obesity or less-active populations.

In addition, dietary habits were not available in this dataset, although they are important determinants of weight loss and maintenance [39,40]. Many other factors, such as metabolic-related variables, general health conditions, and genetic predisposition, can also influence weight changes [41-43]. Comorbidities common in older adults, such as cancer, dementia, or other chronic conditions, were not available in the dataset, but they might also influence physical activity levels and weight change [44,45].

Finally, step count data capture the amount of movement but might not fully reflect the intensity or type of physical activity (eg, step-based measures might underestimate energy expenditure under certain stride frequencies or fail to encompass nonwalking activities such as cycling, running, or standing) [46-49]. Therefore, physical activity effects on weight might be underestimated in this study.

Maximizing ecological validity by linking and considering additional user characteristics, including dietary, metabolic, genetic, comorbidity, and physical activity intensity data, will be necessary for future studies.

### Conclusions

This study revealed effects on weight loss of the trajectories of long-term step counts, as monitored using the “Asmile” mHealth app. Particularly, the long-term trajectory of increased step counts significantly affected 3% weight loss. These findings suggest that sustained engagement in physical activity might play an important role in long-term health management.

## Supplementary material

10.2196/80339 Multimedia Appendix 1 Percentages of participants belonging to latent classes in each latent class mixed model.

10.2196/80339 Multimedia Appendix 2 Means of posterior probabilities belonging to a latent class in each latent class mixed model.

10.2196/80339 Multimedia Appendix 3 Bayesian information criterion at each latent class mixed model.

10.2196/80339 Multimedia Appendix 4 Forest plot of odds ratios of each trajectory for weight loss in sensitivity analysis.

10.2196/80339 Multimedia Appendix 5 List of odds ratios including adjustment variables.

10.2196/80339 Checklist 1 STROBE checklist.

## Abbreviations

CVD: cardiovascular disease
LCMM: latent class mixed model
mHealth: mobile health
SHC: specific health checkup
STROBE: Strengthening the Reporting of Observational Studies in Epidemiology
