# Supplementary material for: One-Year Trajectory of Step Counts and Weight Loss in Adults With Overweight/Obesity: Retrospective Cohort Study
Source: JMIR Mhealth Uhealth. 2026 May 4;14:e80339. doi: 10.2196/80339 (PMC13138716; doi:10.2196/80339)
Supplement: Multimedia Appendix 1 [file mhealth-v14-e80339-s001.docx]

**Multimedia Appendix 1**

Percentages of participants belonging to latent classes in each latent class mixed model

|  | Class 1 | Class 2 | Class 3 | Class 4 | Class 5 |
| --- | --- | --- | --- | --- | --- |
| One latent class LCMM | 100 | - | - | - | - |
| Two latent class LCMM | 84.3 | 15.7 | - | - | - |
| Three latent class LCMM | 13.8 | 77.6 | 8.5 | - | - |
| Four latent class LCMM | 12.8 | 73.6 | 6.9 | 6.7 | - |
| Five latent class LCMM | 8.5 | 4.3 | 10.2 | 71.5 | 5.5 |
