# Supplementary material for: One-Year Trajectory of Step Counts and Weight Loss in Adults With Overweight/Obesity: Retrospective Cohort Study
Source: JMIR Mhealth Uhealth. 2026 May 4;14:e80339. doi: 10.2196/80339 (PMC13138716; doi:10.2196/80339)
Supplement: Multimedia Appendix 2 [file mhealth-v14-e80339-s002.docx]

**Multimedia Appendix 2**

Means of posterior probabilities belonging to a latent class in each latent class mixed model

|  | Class 1 | Class 2 | Class 3 | Class 4 | Class 5 |
| --- | --- | --- | --- | --- | --- |
| One latent class LCMM | - | - | - | - | - |
| Two latent class LCMM | 0.984 | 0.937 | - | - | - |
| Three latent class LCMM | 0.929 | 0.977 | 0.953 | - | - |
| Four latent class LCMM | 0.935 | 0.973 | 0.898 | 0.958 | - |
| Five latent class LCMM | 0.876 | 0.965 | 0.919 | 0.967 | 0.945 |
