# Supplementary material for: One-Year Trajectory of Step Counts and Weight Loss in Adults With Overweight/Obesity: Retrospective Cohort Study
Source: JMIR Mhealth Uhealth. 2026 May 4;14:e80339. doi: 10.2196/80339 (PMC13138716; doi:10.2196/80339)
Supplement: Multimedia Appendix 3 [file mhealth-v14-e80339-s003.docx]

**Multimedia Appendix 3**

Bayesian information criterion at each latent class mixed model

|  | BIC |
| --- | --- |
| One latent class LCMM | 1231498 |
| Two latent class LCMM | 1224289 |
| Three latent class LCMM | 1219651 |
| Four latent class LCMM | 1217810 |
| Five latent class LCMM | 1216244 |
