# Supplementary material for: One-Year Trajectory of Step Counts and Weight Loss in Adults With Overweight/Obesity: Retrospective Cohort Study
Source: JMIR Mhealth Uhealth. 2026 May 4;14:e80339. doi: 10.2196/80339 (PMC13138716; doi:10.2196/80339)

**Multimedia Appendix 4**

Forest plot of odds ratios of each trajectory for weight loss in sensitivity analysis


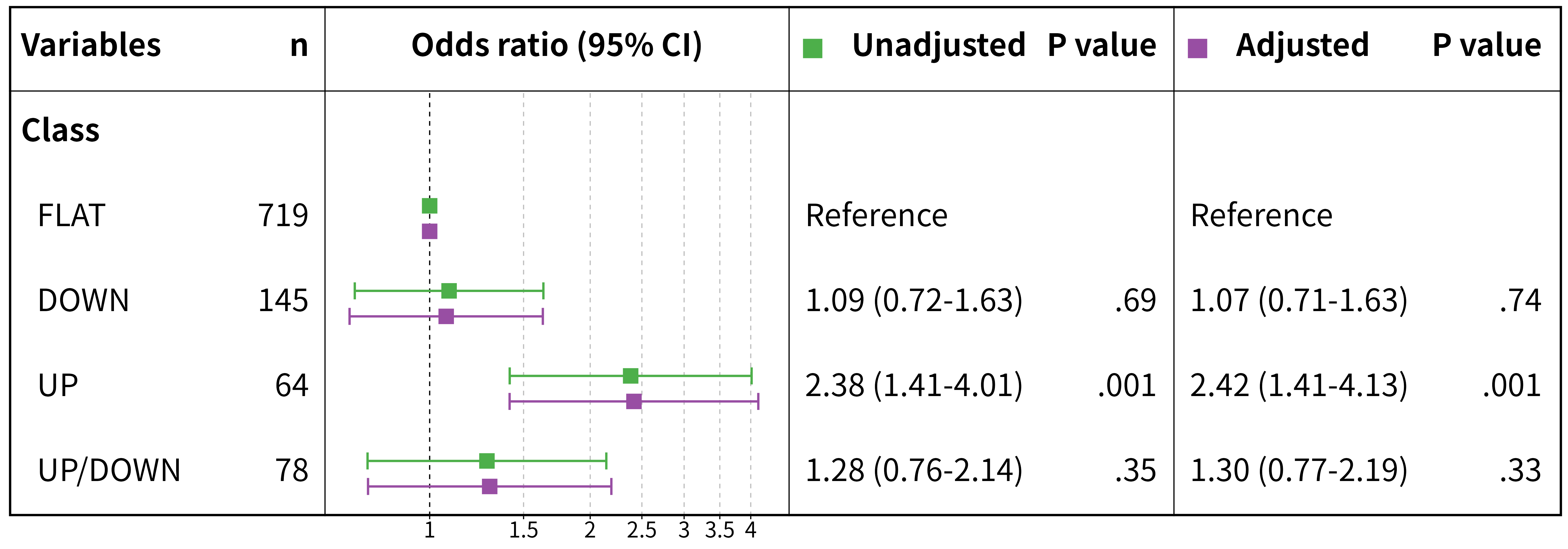

Supplement: Multimedia Appendix 4 [file mhealth-v14-e80339-s004.docx]
