# Supplementary material for: One-Year Trajectory of Step Counts and Weight Loss in Adults With Overweight/Obesity: Retrospective Cohort Study
Source: JMIR Mhealth Uhealth. 2026 May 4;14:e80339. doi: 10.2196/80339 (PMC13138716; doi:10.2196/80339)
Supplement: Multimedia Appendix 5 [file mhealth-v14-e80339-s005.docx]

**Multimedia Appendix 5**

List of odds ratios including adjustment variables

| Variables | Odds ratio (95% CI) | *P* value |  |
| --- | --- | --- | --- |
| CLASS |  |  |  |
| FLAT | Reference |  |  |
| DOWN | 0.92 (0.69-1.22) | .56 |  |
| UP | 2.45 (1.78-3.38) | <.001 |  |
| UP/DOWN | 1.12 (0.79-1.59) | .54 |  |
| Sex |  |  |  |
| Men | Reference |  |  |
| Women | 1.37 (1.13-1.65) | .001 |  |
| Age | | 0.99 (0.98-1.00) | .051 |
| Mean step counts | | 1.01 (0.98-1.04) | .37 |
| Smoking status |  |  |  |
| No | Reference |  |  |
| Yes | 1.49 (1.07-2.05) | .02 |  |
| Medication status |  |  |  |
| No | Reference |  |  |
| Yes | 0.82 (0.68-0.99) | .04 |  |
